# Assessment of Arctic sea ice simulations in cGENIE model and projections under RCP scenarios

**DOI:** 10.1038/s41598-024-67391-1

**Published:** 2024-07-18

**Authors:** Di Chen, Min Fu, Xin Liu, Qizhen Sun

**Affiliations:** 1 Department of Earth, Atmospheric, and Planetary Sciences, Purdue University, West Lafayette, IN USA; 2 National Marine Environmental Forecasting Center, Beijing, China; 3 Hohai University, Nanjing, China

**Keywords:** Simulation skill, Arctic sea ice, Future projection, RCP scenarios, Environmental sciences, Ocean sciences

## Abstract

Simulating and predicting Arctic sea ice accurately remains an academic focus due to the complex and unclear mechanisms of Arctic sea ice variability and model biases. Meanwhile, the relevant forecasting and monitoring authorities are searching for models to meet practical needs. Given the previous ideal performance of cGENIE model in other fields and notable features, we evaluated the model’s skill in simulating Arctic sea ice using multiple methods and it demonstrates great potential and combined advantages. On this basis, we examined the direct drivers of sea-ice variability and predicted the future spatio-temporal changes of Arctic sea ice using the model under different Representative Concentration Pathways (RCP) scenarios. Further studies also found that Arctic sea ice concentration shows large regional differences under RCP 8.5, while the magnitude of the reduction in Arctic sea ice thickness is generally greater compared to concentration, showing a more uniform consistency of change.

## Introduction

As global warming has increased in recent decades, the climate of the Arctic has changed accordingly^1^. Due to the Arctic amplification effect, the Arctic has become the most dramatically changed region of the global climate system, with a warming rate almost four times faster than the global mean^2^. As one of the key indicators of regional climate change and its important role in regulating the climate at middle and high latitudes, sea ice changes in the Arctic region are of great interest. In addition, changes in Arctic sea ice may have implications for the region’s ecosystems, resource exploration, and navigational safety^3–5^. Recent studies show that the Arctic sea ice area in July 2020 fell to its smallest value during the 40-year satellite observations. The Arctic Ocean could experience complete sea ice-free conditions every summer between 2044 and 2067 due to anthropogenic climate change^6^.

Previously, extensive research has been conducted to explore the mechanisms affecting Arctic sea ice, such as sea surface temperature^7,8^ (SST), carbon dioxide^9,10^, local wind forcing^11,12^, cloud effects^13^, surface air temperature^14^ (SAT), oceanic heat transport to the Arctic^15^, Arctic Oscillation^16,17^ (AO), North Atlantic Oscillation^18^ (NAO), Greenland High^19^, other atmospheric pressure patterns, related circulation and energy transports^20,21^, tropical teleconnections^22,23^, volcanic forcing^24^, Internal drivers or variability^25,26^ and other factors^27–30^. However, it is still inconclusive.

Moreover, Arctic sea ice changes are characterized by strong asymmetries and regional variations^31^, such as the Barents sea and the Kara Sea areas, which are the most significant in terms of sea ice loss due to being in the region of the most intense Arctic warming^32,33^, and are strongly associated with local climate change and the occurrence of many extreme weather events, such as AO, NAO, blocking high pressure, extratropical cyclone, blizzard^34–36^.

Given the characteristics and critical contribution of Arctic sea ice change to climate, human production, and life, whether it can be accurately predicted is a hot topic of concern. Yet, due to the discontinuity of the observation data, the uncertainty of the mechanism of the sea ice change and the bias of models, Simulating and predicting changes in Arctic sea ice accurately remain difficult so far^37–39^. At the same time, relevant sea ice monitoring departments and early warning organizations have been looking for models to meet their practical needs. Subject to a variety of constraints, the model not only needs to provide timely information feedback, but also has the characteristics of easy transplantation, simple operation, cost-effectiveness, and computational efficiency. Given these, cGENIE.muffin Earth system model with its unique features, has demonstrated great potential in simulation sea ice since the simulation effect of the model is ideal in many fields^40–42^, but there is a lack of research on Arctic sea ice using this model.

Here, we use multiple methods to evaluate the skill of cGENIE.muffin model to simulate sea ice under Representative Concentration Pathways (RCPs). Combining previous research and our preliminary analysis. We hypothesize that the model in overall has high skill in simulating Arctic sea ice, and can make reasonable predictions of future sea ice changes. This work will provide a theoretical basis and reference for the relevant polar environment monitoring and forecasting departments.

### Model simulation skill assessment

**Figure 1:**
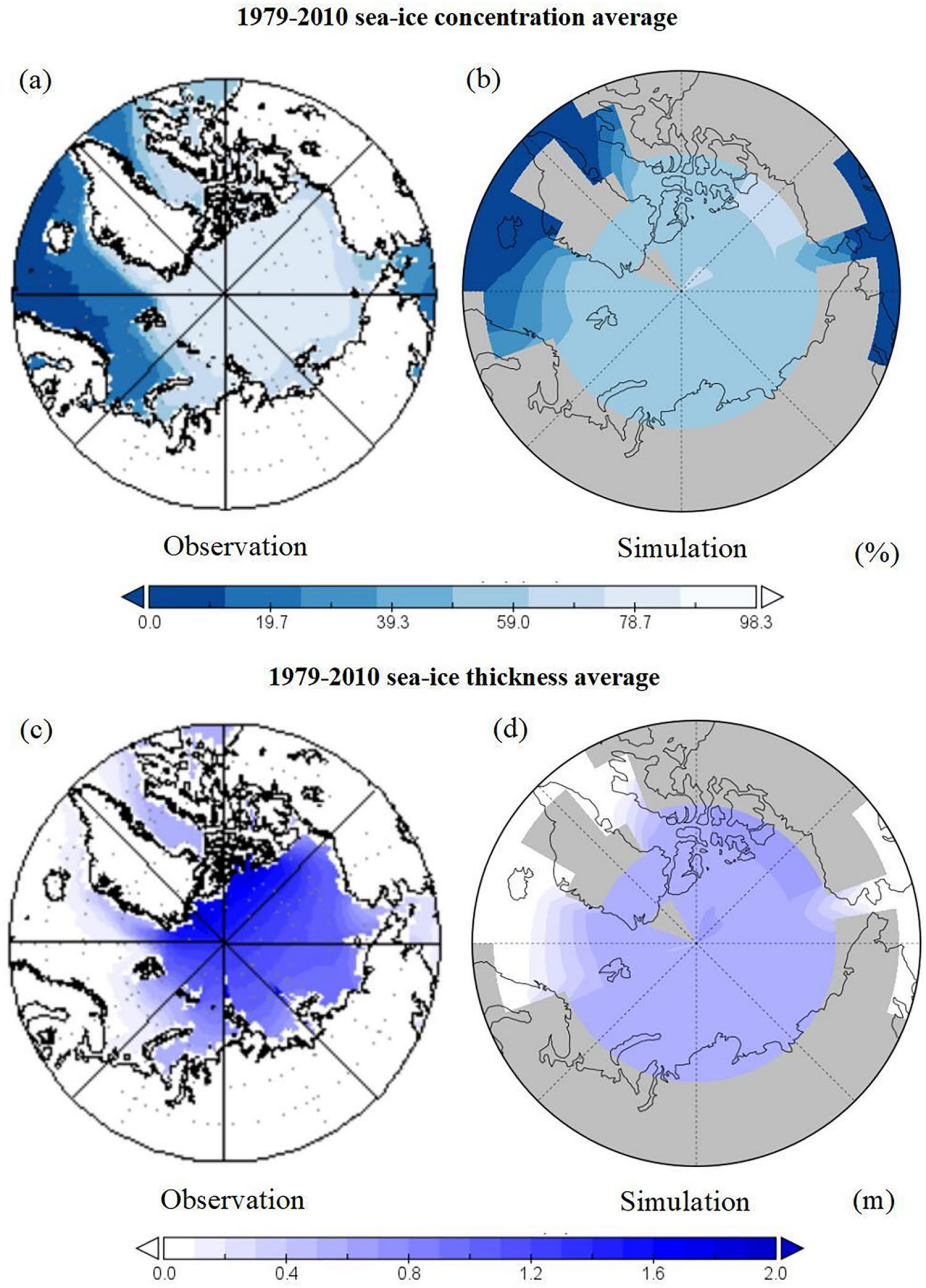
The climatology of simulation and observation from 1979 to 2010. The left is the observation (sea ice concentration (SIC) from the Hadley center; sea ice thickness (SIT) from GIOMAS and the right is the cGENIE model simulation results. (**a,b**) Are SIC; (**c,d**) are SIT. Unit in% and meters, respectively.

To project future changes in Arctic sea ice using cGENIE.muffin model, it is critical to first fully evaluate the model’s simulation capability. Considering the accuracy of the observational data and the convenience of referring to the results of previous related studies, we compare the simulation results of the Arctic sea ice from 1979 to 2010 using the model with the observational data (Fig. 1).

A comparison of Arctic SIC and SIT from observations and model simulations for 1979–2010 is given in Fig. 1. From the observations, it can be seen that the SIC (Fig. 1a) and SIT (Fig. 1c) have maximum values in the Arctic Ocean, the Sea of Okhotsk, the Barents Sea, and the Kara Sea, and minimum values in the Labrador Sea, the North Atlantic subpolar region, and the Hudson Bay, etc. The gradients are largest in the Greenland Sea, the Norwegian Sea, the Baffin Bay, and the Chukchi Sea, and they decrease toward the North Atlantic and the North Pacific.

Specifically, for the sea-ice concentration simulation, the model captures the spatial distribution of sea-ice variability reasonably, but there are significant discrepancies at the largest gradients in the Greenland Sea, Norwegian Sea, Baffin Bay, and the Chukchi Sea, with a maximum deviation of 35.6%; and there is also a large bias near the Beaufort Sea, with a maximum deviation of more than 50%; The SIT simulation is better than the concentration simulation, but still has a large discrepancy at the largest sea ice gradient and in the Barents Sea, with a maximum deviation of 0.5 m.

In addition, muffin also has good simulation ability in the temporal distribution of sea ice compared with the observed data, the overall trends of SIC and SIT are basically the same, with correlation coefficients of 0.56 and 0.62, respectively (P < 0.01, figure omitted).

In general, the overall simulation effect is good, except for some obvious differences in certain regions. Here, in order to accurately measure the simulation skill of the model and establish the basis for the next step, we use the observation data in combination with the historical data of CMIP5 to further quantify the simulation skill of the Arctic SIC simulated by cGENIE.muffin model based on the methods adopt in the prior investigation^43^ (Table 1), which are commonly used in assessing sea-ice models and have high reliability^44–46^ (see “Methods” section). Furthermore, to comprehensively compare the overall performance of the model in terms of its skill in simulating SIC compared to other CMIP5 models, we also inherit the comprehensive scoring criterion, NF-score, which takes into account the synergistic effects of sea ice simulation in the the Barents Sea and the Kara Sea (BK) and other Arctic regions (exBK) with different weights based on the strong feedback of BK seas to Arctic climate and its interconnection with mid-latitude climate compare to other areas. Skill (Sk) values were calculated in each grid, and the final skill value was obtained after averaging. A higher skill value indicates better model performance (see “Methods” section). To bridge the gap between relative error and skill score, the residual relative error (RRE) (one minus the absolute value of relative error) was used instead of the relative error itself. For exBK and BK regions, four values (RRE_exBK_, SK_exBK_, N_RRE_BK_ and N_SK_BK_) were obtained. Here, the weight coefficients of four factors including sea ice trends of the exBK, sea ice trends of the BK, sea ice anomalies of the exBK, and sea ice anomalies of the BK were 0.1, 0.3, 0.2 and 0.4, respectively. i.e., the NF-score can be calculated by adding these four weighted scores and normalizing it. The formula can be written in the following form:

$${\text{NF-score }} = {\text{ Normalized }}[ 0.1 \, \times {\text{ N}}\_{\text{RRE}}_{{{\text{exBK}}}} { + } 0.2 \times {\text{ N}}\_{\text{SK}}_{{{\text{exBK}}}} { + } 0.3 \times {\text{ N}}\_{\text{RRE}}_{{{\text{BK}}}} { + } 0.4 \times {\text{ N}}\_{\text{SK}}_{{{\text{BK}}}} ].$$

**Table 1:** Model ranking according to the NF-score.

Ranking	Model name	N_RRE_BK_	N_RRE_exBK_	N_SK_BK_	N_SK_exBK_	NF-score
1	MIROC-ESM CHEM	0.85	0.25	1.05	2.11	1.87
2	MPI-ESM-MR	1.04	0.49	1.20	0.29	1.49
3	NorESM1-M	0.24	0.86	0.84	0.65	1.04
4	CNRM-CM5	− 0.10	0.49	1.05	0.37	0.85
5	IPSL-CM5A-MR	0.71	− 0.14	0.47	− 0.09	0.62
6	FGOALS-s2	1.35	− 0.71	− 0.55	0.61	0.40
7	GISS-E2-R p1	0.81	0.12	0.12	− 0.72	0.26
8	CanESM2	1.14	− 0.84	− 0.17	− 0.98	− 0.01
9	ACCESS1-3	− 1.00	− 0.25	0.37	− 0.04	− 0.31
10	BCC-CSM1-1-M	0.43	− 0.95	0.00	− 1.31	− 0.38
11	cGENIE.muffin	− 1.09	0.21	0.32	− 1.58	− 0.47
12	MIROC-ESM	− 2.41	0.75	− 0.18	2.00	− 0.53
13	GFDL-ESM2M	− 1.92	0.04	0.41	− 0.01	− 0.68
14	IPSL-CM5A-LR	− 0.72	− 0.51	− 0.72	0.38	− 0.79
15	MRI-CGCM3	− 1.02	1.16	− 0.50	− 0.76	− 0.90
16	FGOALS-g2	− 0.53	0.90	− 1.37	− 1.57	− 1.55

N_RRE represents the normalized residual relative error for BK and exBK trends, and N_Sk represents the normalized skill for BK and exBK anomalies, respectively. NF-score can be calculated from the weighted integral of four components (N_RREs and N_Sks) and normalizing it. The model list is shown in descending order in terms of NF score^43^.

The results of the corresponding comparisons of the observed data with the CMIP5 models and cGENIE.muffin model, respectively, are shown in Table 1, and the results are shown in descending order according to the NF score.

From the table, although the model does not rank high in comparison with other CMIP5 models, considering the excellent performance and reputation of other models in sea ice simulation, we have reason to believe that the model has good sea ice simulation skills; thus, we can infer that our results are in line with the initial hypothesis.

### The potential contribution of different drivers to Arctic sea ice change

As we mentioned before, despite a lot of previous work that has been done, the mechanism of Arctic sea ice change is still uncertain. Therefore, we will use the results of cGENIE.muffin model to explore the relationship between Arctic sea ice and some of these drivers. Since sea ice is formed by the freezing of seawater, sea surface temperature, salinity, and near-surface air temperature are all important indicators to characterize the sea ice information; in addition, considering the important role that greenhouse gases play in the RCP, the global pCO_2_ will also be taken into account.

**Figure 2:**
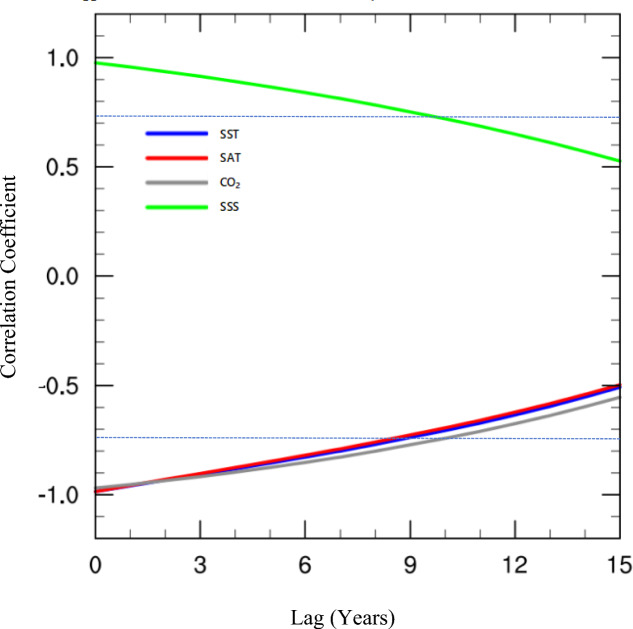
The lag correlation of SIC with multiple drivers (SST, SAT, CO2, Sea Surface Salinity (SSS)); blue, red, gray and green curves represent SST, SAT, CO2, SSS, respectively. The dash line represents the 99% confidence level.

Based on the previous studies and the reality of cGENIE.muffin model, we show the lagged correlations between Arctic SIC and the drivers (Fig. 2); it can be seen that SST, SAT, and CO2 are negatively correlated with the sea ice, and all of them are most obviously correlated during the simultaneous period, with correlation coefficients of − 0.96 (P < 0.01). The correlation coefficients of these three drivers within 9 years of the sea ice lag are all significant, indicating that the effects of SST, SAT, and CO2 on Arctic sea ice have strong interannual characteristics; meanwhile, sea surface salinity (SSS) and Arctic sea ice are positively correlated, and all of them are most obviously correlated during the simultaneous period, with correlation coefficients of 0.92 (P < 0.01), and then weakened gradually over time; Meanwhile, SSS also has significant interannual effects on sea ice.

### Future projections of Arctic sea ice

**Figure 3:**
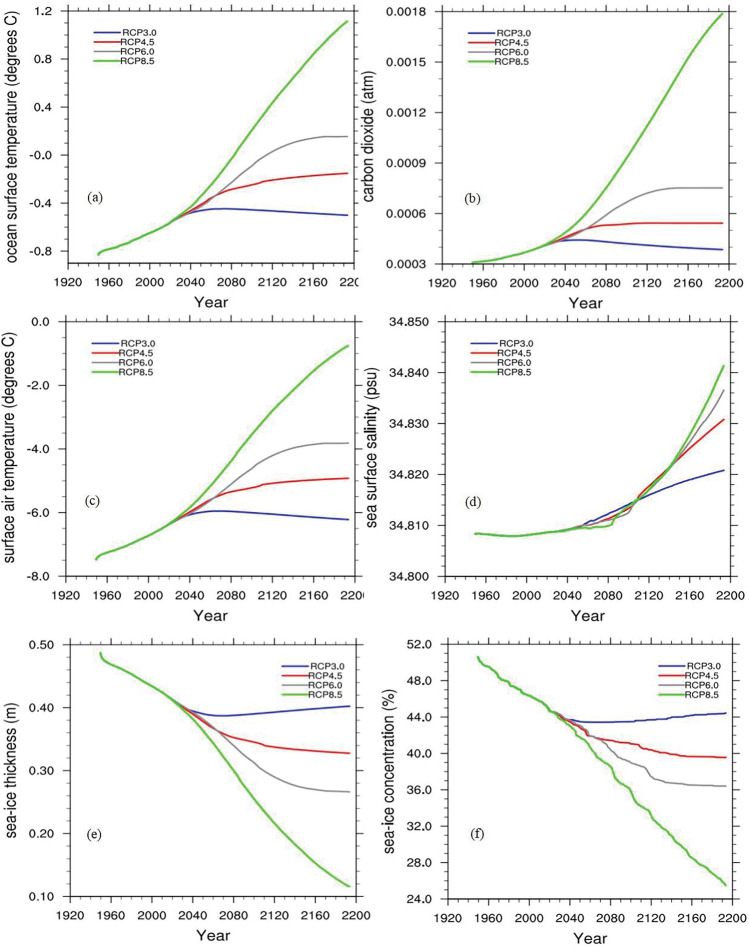
Time series of Arctic sea ice and drivers variations from 1950 to 2100 (SST, SAT, CO2, Sea Surface Salinity (SSS)); blue, red, gray, and green curves represent RCP3.0,4.5,6.0 and 8.5 respectively. The top row (**a, b**) is SST and CO2 respectively; the center (**c, d**) is SAT and SSS; and the bottom (**e, f**) is SIT and SIC.

Given the promising simulation skill of cGENIE.muffin model for Arctic sea ice, we use this model to predict the Arctic sea ice under different future emission scenarios. Figure 3 shows the drivers of sea ice changes and the changes in Arctic sea ice over time under different RCP scenarios (RCP3.0, RCP4.5, RCP6.0, RCP8.5).

It can be observed that under RCP3.0, RCP4.5, and RCP6.0 scenarios, the growth of SST, SAT, and CO2 is relatively smooth. Around 2040, noticeable differences begin to emerge, and these differences gradually amplify over time; while under RCP8.5, the growth of these drivers is significantly much higher than that of the other scenarios. However, the situation of SSS is somewhat different, i.e., the difference in change across the four scenarios is small and the growth is the smallest among these drivers, which, despite the relatively small change, still has an impact that cannot be ignored (Fig. 3a–d). Since the differences in these drivers are so pronounced, how will Arctic sea ice change under their influence? Fig. 3e,f present future projections of Arctic SIC and SIT, respectively; similarly, both show negative trends of varying magnitude, with a clear watershed around 2040, and the differences are large under different RCP scenarios. Specifically, Arctic SIT decreases to a minimum around 2050 under RCP3.0, and then increases slowly and consistently, recovering to 2020 levels around 2100; it continues to decrease by varying amounts under both RCP4.5 and RCP6.0 scenarios, and it is least optimistic under RCP8.5, i.e., it continues to decrease under the business as usual scenario, Arctic SIT will show an alarming rate of decrease, down to 0.14 m in 2160 compared to 0.48 m in 1950, a decrease of more than 70%, and will continue to decrease rapidly at the same rate. Likewise, future changes in Arctic SIC show similar variability, except that they show more annual fluctuations compared to SIT; the rate of concentration reduction in the RCP8.5 scenario is still striking, dropping to about 28% in 2160 compared to 50% in 1950, a reduction of up to 44%.

**Figure 4:**
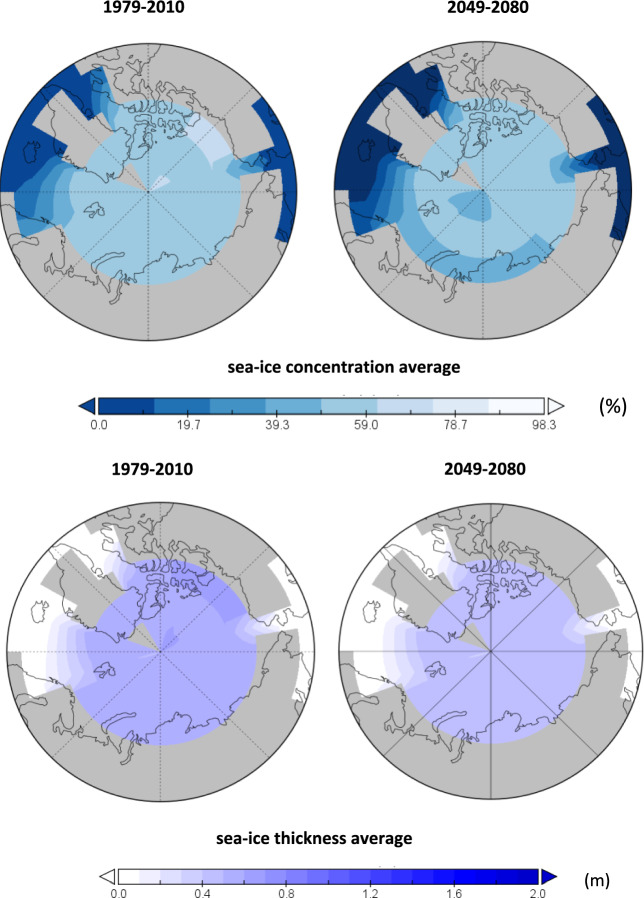
The projections of Arctic SIC and SIT compared to historical data using the cGENIE.muffin model under the RCP8.5 scenario. The left shows projections for 2049–2080 and the right shows historical simulations for 1979–2010. The top represents SIC and the bottom is SIT. Unit in% and meters, respectively.

Given that the magnitude of sea ice change under different RCP scenarios does not show large differences until after 2040, and also considering the possibility that the RCP scenarios underestimate the effects of global warming^47^, we further show the spatial changes in Arctic sea ice from 2049 to 2080 under RCP8.5 (Fig. 4). Compared with 1979–2010, the Arctic SIC in 2049–2080 will decrease in most areas, and the large value of the decrease is mainly concentrated in the Barents sea, Kara Sea, Laptev Sea, and Beaufort Sea, with a decrease of about 20–30%, locally more than 40%; followed by the Greenland Sea, Norwegian Sea and Chukchi Sea, with a reduction of about 10–20%; Meanwhile, the future spatial distribution of Arctic SIT will be more pessimistic, the Arctic SIT in 2049–2080 compared to 1979–2010 have a significant decrease over almost all regions, showing more uniform regional changes. The decrease is mainly concentrated in Baffin Bay and Beaufort Sea, with a decrease of about 50–60%, locally more than 70%.

In short, under RCP8.5, the future changes of both Arctic SIC and SIT show obvious temporal and spatial decreasing trends, but there are some obvious differences; i.e., the concentration of Arctic sea ice, although the overall decrease is smaller than that of SIT, shows larger regional differences; while the SIT of Arctic sea ice, although the decrease is generally larger than that of concentration, presents a more uniform consistency of change across the region.

## Discussion

Through our analysis, we found that cGENIE.muffin model can simulate the concentration and thickness of Arctic sea ice well; the new scoring criteria developed by us further found that the model is superior to some of the CMIP5 models, and shows a strong potential for sea ice prediction, which also verifies that our initial hypothesis is correct. Further, we use the model to explore some of the drivers of sea ice changes, and find that SST, SAT, and CO2 are all negatively correlated with sea ice, with correlation coefficients of − 0.96 (P < 0.01), and have strong interannual variability. In contrast, SSS is positively correlated with Arctic sea ice, with correlation coefficients of 0.92 (P < 0.01), and also has significant interannual effects. On this basis, under different RCP scenarios, we use the model to predic Arctic sea ice, and find that they show different trends and magnitudes, and the concentration and thickness of Arctic sea ice under the influence of these show different degrees of decreasing trends, with a clear watershed near 2040, and with large differences under different RCP scenarios. Among them, the RCP8.5 scenario has the most obvious rate of their decline; the thickness of Arctic sea ice will drop to 0.14 m in 2160 compared to 0.48 m in 1950, and the decrease will be more than 70%; the Arctic SIC will drop to about 28% in 2160 compared to 50% in 1950, and the decrease will be as high as 44%. In addition, further research also found that under the business as usual scenario, the future changes of both Arctic SIC and SIT show obvious temporal and spatial decreasing trends, but there are some obvious differences; that is, the Arctic SIC, although the overall decrease is smaller, shows a larger regional difference; and the Arctic SIT, although the overall decrease is larger than that of concentration, shows a more consistent region-wide consistency of change; the mechanism behind these interesting features of change is still unclear and will be subject to in-depth investigation in our following work.

In summary, in the context of global warming, the extent of Arctic sea ice will continue to decline, and SIT will become thinner. Suppose effective energy saving and emission reduction measures are not taken. In that case, the Arctic sea ice under the future business-as-usual scenario will continue to decrease at an alarming rate, and the rate of decrease in SIT will be significantly higher than that of sea ice extent. In addition, cGENIE.muffin model has reasonable simulation performance, relatively simple design, convenient transplantation, efficient operation. Given the multiple pressures of inadequate basic research resources in recent years, these features together show great potential of cGENIE.muffin model for sea ice simulation. Our work will provide not only a reference for the operational forecasting department but also a theoretical basis for the relevant governmental departments in future disaster prevention and mitigation, ecological protection, and navigation safety.

## Methods

### Data

We use Arctic sea ice concentration data from the Hadley Center with a horizontal resolution of 1.0° × 1.0°^47^ from 1871 to the present to assess the simulation skills of cGENIE.muffin model. This data comes from a variety of sources including ground-based, ship-based, and model-based, making it one of the most credible sea ice observations since the introduction of satellite-based passive microwave retrieval in 1979. The Arctic sea ice thickness data comes from the Global Ice-Ocean Modeling and Assimilation System (GIOMAS) with a mean resolution of 22 km (https://psc.apl.washington.edu/zhang/Global_seaice/).

We select the area of 65°–90° N as the area of the study, which contains the Greenland Sea and Norwegian Sea, Barents Sea, Kara Sea, Okhotsk Sea, Labrador Sea, and Chukchi Sea. In addition, to increase the robustness of the results, we also adopt the fifth phase of the Coupled Model Intercomparison Project^48^ (CMIP5) to quantify further the simulation skill of sea ice (http://pcmdi3.llnl.gov/esgcet/home.htm). Given the availability of relevant data, completeness, and the performance of related patterns in previous related studies, we selected 15 of them (see Table 1). Historical simulations of these data were from 1979 to 2005 and extended from 2006 to 2010 using Representative Concentration Pathways 8.5 (RCP 8.5, which represents a “business as usual” scenario) simulation data, consistent with the general treatment of similar studies.

Prior to our analyses, both observations and model results were regrided into the same horizontal resolution through linear interpolation.

### cGENIE.muffin Earth system model

We use the open-source intermediate-complexity Earth system model cGENIE (“muffin” release) to investigate and verify our hypothesis^49^. A manual detailing code installation, basic model configuration, tutorials covering various aspects of model configuration and experimental design, and the output and processing of results are assigned the following 10.5281/zenodo.4615662.

### Evaluation of model simulation skills

The detailed processes of quantification are as follows: firstly, the fraction of the grid cell covered by SIC was multiplied by the area of grid cell to calculate sea ice area (SIA) for BK (70.5°–81.5° N, 19.5°–100.5° E) and exBK regions, respectively (as shown in Eq. (1):

1 $$\begin{aligned} {\text{SIA}}_{{{\text{BK}}}} = & \int\limits_{{70.5^{ \circ } \;{\text{N}}}}^{{81.5^{ \circ } \;{\text{N}}}} {\int\limits_{{19.5^{ \circ } \;{\text{E}}}}^{{100.5^{ \circ } \;{\text{E}}}} {2\pi r^{2} \left( {\sin \left( {\frac{\varphi + 1}{{180}}\pi } \right) - \sin \left( {\frac{\varphi }{180}\pi } \right)} \right)} \frac{{{\text{SIC}}\left( {\lambda ,\varphi } \right)}}{360}} {\text{d}}\lambda {\text{d}}\varphi , \\ {\text{SIA}}_{{{\text{exBK}}}} = & \int\limits_{{40.5^{ \circ } \;{\text{N}}}}^{{89.5^{ \circ } \;{\text{N}}}} {\int\limits_{{179.5^{ \circ } \;{\text{W}}}}^{{179.5^{ \circ } \;{\text{E}}}} {2\pi r^{2} \left( {\sin \left( {\frac{\varphi + 1}{{180}}\pi } \right) - \sin \left( {\frac{\varphi }{180}\pi } \right)} \right)} \frac{{{\text{SIC}}\left( {\lambda ,\varphi } \right)}}{360}} {\text{d}}\lambda {\text{d}}\varphi - {\text{SIA}}_{{{\text{BK}}}} , \\ \end{aligned}$$

where the earth radius r = 6 731 km.

Then, their linear trends were estimated using the least square method. Comparing the SIA trends of model outputs with observations, relative errors of the trends (Eq. 2) were calculated. A lower absolute value of the relative error indicates a better performance of the models.

2 $${\text{Relative}}\;{\text{error}} = \left| {\frac{{X_{\bmod } - X_{{{\text{obs}}}} }}{{X_{{{\text{obs}}}} }}} \right|,$$

where Xmod and Xobs represent the modeled and observational SIA trends, respectively.

Secondly, detrended SIC anomaly time series were obtained for each grid, with both climatology and linear trend being subtracted from the original data. A quantitative comparison between the model results and observations was conducted using the method below^50^ (Eq. 3):

3 $${\text{Skill}} = 1 - \begin{array}{*{20}c} {\frac{{_{i = 1}^{N} \left| {X_{\bmod } - X_{{{\text{obs}}}} } \right|^{2} }}{{\sum\nolimits_{i = 1}^{N} {\left( {\left| {X_{\bmod } - \overline{X}_{{{\text{obs}}}} } \right| + \left| {X_{{{\text{obs}}}} - \overline{X}_{{{\text{obs}}}} } \right|} \right)^{2} } }}} \\ \\ \end{array},$$

where X represents the variable, X̅ represents its time mean, and the subscripts mod and obs represent model results and observations, respectively. Skill (Sk) values were calculated in each grid, and the final skill value was obtained after averaging. A higher skill value indicates better model performance.

### Residual relative error

Residual relative error^51^ (RRE) is the most commonly used statistical indicator, which reflects the average situation of the variance obtained between the pattern simulation and the actual observed value, which is non-negative, and the higher the value indicates the higher the skill.

$${\text{RRE }} = { 1} - {\text{Relative error}}$$

## Data Availability

The ice thickness distribution data from the Norwegian Polar Data Centre, https://data.npolar.no/dataset/b94cb848-3120-4f29-a827-298108e0d059. The sea ice concentration data used are available at https://www.metoffice.gov.uk/hadobs/hadisst2/. The CMIP5 data is available at https://esgf-node.llnl.gov/projects/cmip5.

## Code availability

The code that supports the findings of this study is available from the corresponding author upon reasonable request.
